# Computational Fluid Dynamics Modeling of Top-Down Digital Light Processing Additive Manufacturing

**DOI:** 10.3390/polym15112459

**Published:** 2023-05-26

**Authors:** Hesam Moghadasi, Md Tusher Mollah, Deepak Marla, Hamid Saffari, Jon Spangenberg

**Affiliations:** 1 School of Mechanical Engineering, Iran University of Science and Technology (IUST), Narmak, Tehran 16846-13114, Iran; 2 Department of Mechanical Engineering, Technical University of Denmark, Kgs, Lyngby 2800, Denmark; 3 Department of Mechanical Engineering, Indian Institute of Bombay, Mumbai 400076, India

**Keywords:** additive manufacturing (AM), computational fluid dynamics (CFD), digital light processing (DLP), vat photopolymerization, stability time

## Abstract

Digital light processing (DLP) as a vat photopolymerization technique is one of the most popular three-dimensional (3D) printing methods, where chains are formed between liquid photocurable resin molecules to crosslink them and solidify the liquid resin using ultraviolet light. The DLP technique is inherently complex and the part accuracy depends on the process parameters that have to be chosen based on the fluid (resin) properties. In the present work, computational fluid dynamics (CFD) simulations are presented for top-down DLP as photocuring 3D printing. The effects of fluid viscosity, travelling speed of build part, travelling speed ratio (ratio of the up-to-down traveling speeds of build part), printed layer thickness, and travel distance considering 13 various cases are scrutinized by the developed model to obtain a stability time of fluid interface. The stability time describes the time it takes for the fluid interface to show minimum fluctuations. According to the simulations, a higher viscosity leads to prints with higher stability time. However, lower stability times in the printed layers are caused by a higher traveling speed ratio (TSR). The variation in settling times with TSR is extremely small in comparison to that of viscosity and travelling speed variations. As a result, a declining trend can be detected for the stability time by increasing the printed layer thickness, while by enhancing the travel distance values, the stability time demonstrated a descending pattern. In total, it was revealed that it is essential to choose optimal process parameters for achieving practical results. Moreover, the numerical model can assist in the optimizing the process parameters.

## 1. Introduction

Additive manufacturing (AM) is a family of processes capable of producing three-dimensional (3D) objects. Many AM processes can be subdivided into three steps: first, a 3D model is designed using a computer designing software. Second, the 3D model is cut into slices. Ultimately, the model is printed by a layer-by-layer technique. Therefore, theoretically, AM can be used to make any complex 3D geometry [1,2,3,4]. Because of its advantages, the 3D printing technology is used in various industrial sectors such as energy [5,6], aerospace [7,8], robotics [9,10], food [11,12], chemical [13,14], pharmaceutical [15,16], and biomedical [17,18].

The basic idea of AM was first proposed in the 1980s. Since then, it has rapidly progressed, leading to the development of several technologies. The photocuring approach in AM is one of the earliest techniques in 3D printing, which involves solidification of a photosensitive liquid resin by light exposure. Only the regions irradiated with light become photopolymerized, while the other regions of the resin remain in liquid form. Therefore, the printed models can be separated from the resin quickly and easily. In the photocuring method, models can be printed rapidly with high precision as a result of the fast polymerization rate. Photocuring-based AM includes various methods such as stereolithography (SLA) and digital light processing (DLP) [19,20].

**Figure 1:**
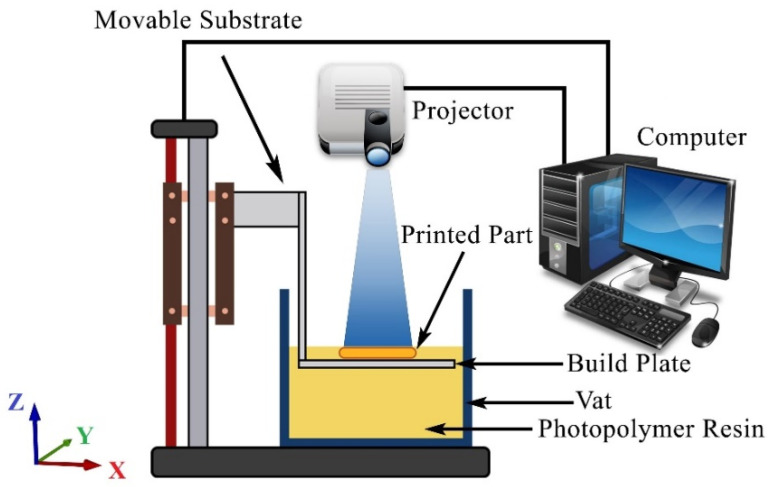
The schematic of the top-down DLP printing process.

DLP is a popular technique for additive manufacturing that involves using liquid photocurable resin molecules to form chains that crosslink and solidify under ultraviolet (UV) light. The top-down DLP printing process is illustrated in Figure 1, where the light source is positioned above the build plate in a manner similar to a projector used for home theaters or office presentations. An array of micromirrors is used to selectively cure a prepolymer resin into the desired geometry by transmitting UV light from the projector. DLP technology plays a crucial role in determining the printing precision and image formation, with the DMD or DLP chip being the key component. This advanced optical switching tool contains two million small microscopes arranged in regular arrays that can project a full digital image onto a screen or surface by coordinating with image signals, digital video, projection lenses, and light sources.

The processes can be performed with either a bottom-up or a top-down technique. The latter is the focus of this paper and it is illustrated for the DLP process in Figure 1. The working principle for the top-down technique is that after a layer has been exposed for light, the build-plate first moves downwards followed by an upwards movement until the right layer thickness is reached. This procedure allows new resin to flow on top of the already cured part of the component, and as soon as the resin has stabilized (i.e., no ripples in the resin surface), a new layer can be generated. The main advantage of a downward-moving platform is that it can be moved very easily due to gravity. Moreover, larger and heavier prints are allowed by top-down printers with no falling off the print bed and less failure for the smoother process. In addition, easier and cleaner workflows are presented by top-down 3D printers.

Gao et al. [21] investigated the bending behavior of hexagonal and square honeycomb sandwich structures of ceramic material fabricated by the top-down DLP approach, experimentally. Their results showed that ceramic samples are sensitive to the defects formed during the printing, sintering, and cleaning processes. Li et al. [22] scrutinized porous β-TCP/BG scaffolds with various solid loading ceramic slurry utilizing the top-down DLP 3D printing device. In their work, the impacts of solid loading on viscosity and curing reactivity were examined. The outcomes demonstrated that the cure depth and overgrowth tended to decrease with the solid loading. Sun et al. [23] developed a top-down DLP 3D printing technique for high-quality translucent alumina ceramic. The curing properties of alumina suspensions were examined by changing the curing depth and polymerization conversion behavior. They proved that DLP AM technology is a feasible technology to produce dense and pore-free alumina ceramics with suitable optical transmittance.

Several works have explored the SLA and DLP [24,25,26,27,28,29]. Li et al. [30] studied theoretical predictions of the DLP working curve for different photocurable substances. They developed an analytical model based on differential analysis to correlate a single layer’s cured thickness and UV light exposure time. This model can save time and reduce resin waste when developing new DLP printing resins. Kadry et al. [31] investigated the feasibility of utilizing DLP 3D printers in making solid oral dosage forms. They also assessed the tablets for mechanical strengths, drug content, swellability, microscopic features, weight variation, drug release profiles, and drug–polymer interactions. They found that by increasing the number of perforations in the tablets, the drug release increased. Moreover, they revealed that DLP 3DP can be utilized as a platform to fabricate oral tablets with different release profiles and well-defined shapes.

Sun and X. Zhang [32] investigated micro SLA experimentally and by numerical modelling. The experimental measurements of curing width and depth were in good agreement with the numerical model. In addition, the model was exploited to study critical process parameters and ultimate fabrication precision. Tarabeux et al. [33] developed a numerical model for resin curing during the SLA procedure while considering the scattering phenomenon. The model was validated by testing a commercial photopolymerizable alumina paste.

The significance of the vat polymerization technologies was stated by former studies based on their applications. By vat polymerization technologies, the best compromise is presented between surface quality and printing resolution, although it is presently indicated that the vat polymerization process chain (particularly, DLP) still includes some open points. It is valuable to assess the process parameters for optimizing the printed layers’ ultimate features and morphological properties. In this paper, a computational fluid dynamics (CFD) model is developed to simulate the resin flow in top-down photocuring AM (i.e., could be either SLA or DLP). The model is used to investigate the effect of various process parameters on the stability time, which is the time it takes from the light exposure ending for one layer and light exposure starting for the subsequent layer. This includes the movement of the build plate and the time it takes for the resin surface to stabilize. The methodology of the study is explained in Section 2. The outcomes are provided and discussed in Section 3. Section 4 deals with the conclusion of the research work.

## 2. Model Description

### 2.1. Governing Equations

The governing equations (Equations (1) and (2)) for the numerical model include the mass and momentum conservation equations of an incompressible Newtonian fluid, respectively:

(1) $$\frac{\partial u_{j}}{\partial x_{j}}=0$$

(2) $$\rho \frac{\partial u_{i}}{\partial t}=-\frac{\partial p}{\partial x_{i}}+\mu \frac{\partial^{2}u_{i}}{\partial x_{j}\partial x_{j}}+\rho g_{i}$$

where $u_{i}$ and $g_{i}$ denote the velocity component and the gravitational body forces per mass unit, both in the i-direction, p is the local pressure, t is the time, $x_{i}$ is the spatial coordinates, and j=1,2,3 is a summation index. In actuality, the first and second RHS terms in Equation (2) are pressure gradient and diffusion term, respectively. For a Newtonian fluid, viscosity operates as a diffusion of momentum. Moreover, the third RHS term is the external/body force term that acts on the fluid (gravitational force). The force of gravity acts on the element as it moves in the vertical direction.

### 2.2. Numerical Model

The 2D CFD model was developed using the commercial software FLOW-3D v.12 update 3 [34]. The software has formerly been successfully utilized for the simulation flow taking into account various kinds of materials [35,36,37,38]. An implicit pressure–velocity solver GMRES (Generalized Minimum Residual) was used to solve the equations for material flow [39,40,41]. Using a second-order accurate scheme in space and an implicit time-discretization, the governing equation was also solved. Additionally, the free surface of the fluid was explicitly advected by the volume of fluid (VOF) technique with a sharp interface reconstruction [42,43].

**Figure 2:**
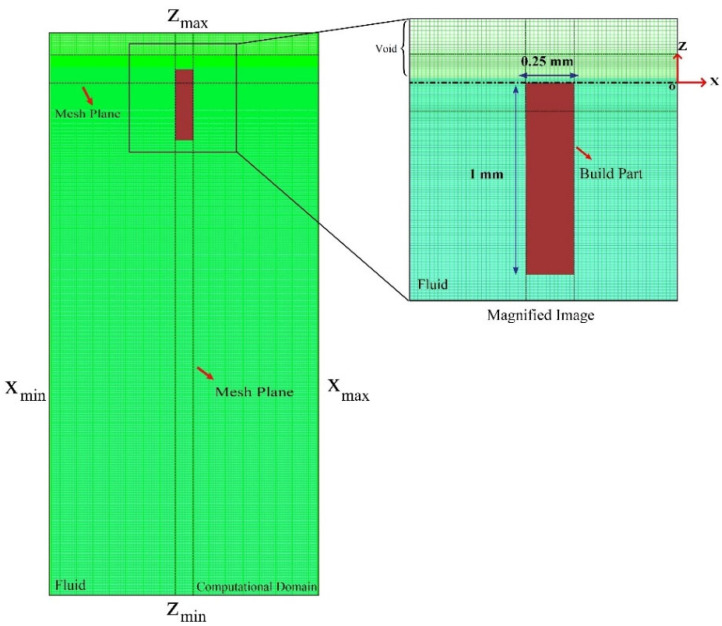
Illustration of model geometry with the computational domain.

Figure 2 shows the model geometry along with the computational domain. Furthermore, $x_{min}$, $x_{max}$, and $Z_{min}$ are assumed as wall boundary conditions while a symmetry boundary is applied at $Z_{max}$. Note that the walls were not in contact with the build plate. In addition, the resin was considered as Newtonian and incompressible fluid and the flow was treated as laminar.

In the present work, a wall was simulated where part of it had already been printed, and a layer was utilized with the height and length of 1 mm and 0.25 mm, respectively, which was located below the origin at t=0. The layer started to flow in the opposite z-direction at time t>0, within the domain with the specific process parameters. Then, the building part moved up in the z-direction to complete the printing process.

Before further assessments, the grid independency was evaluated for 104,091, 68,580, and 33,108 elements under determined conditions (Table 1). Based on the mesh sensitivity analysis, Mesh 2 with 68,580 cells was utilized throughout the research study, since it provided a proper compromise between computational cost and accuracy. Additionally, the mesh was refined near the walls.

### 2.3. Post-Processing

**Figure 3:**
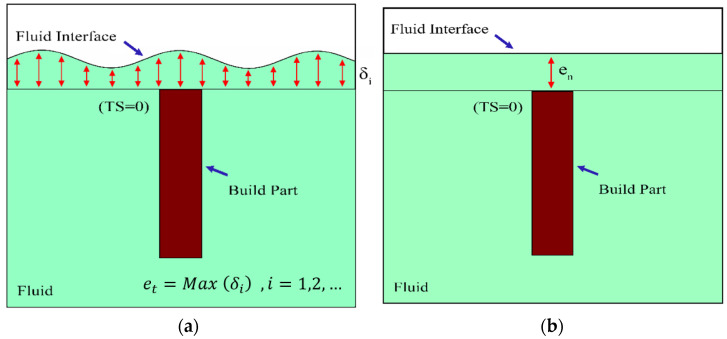
The schematic of post-processing calculation: (**a**) at $t_{m}\leq t\leq t_{s}$, (**b**) at t→∞.

The CFD results were post-processed using MATLAB to obtain the stability time $\left(t_{s}\right)$ of the resin surface at different thickness deviations $∆e_{t}$ of the layer. For each time step between the traveling time ($t_{m}$) and a relatively long time (i.e., finishing time $t_{f}=$ 60 s) with an interval of 0.5 s, the thickness deviations $\left(∆e_{t}\right)$ were determined utilizing the Equation (3).

(3) $$∆e_{t}=\left|e_{t}-e_{n}\right|$$

where $e_{t}$ is the thickness of the layer at a given time and $e_{n}$ represents the nominal thickness of the layer (50 μm). According to Figure 3, $e_{t}$ is determined as the maximum distance of the fluid interface from the surface of the build part before printing the next layer ($\delta_{i}$) where TS=0. For each time step, the distance is determined utilizing the cell fluid fractions values along the z-direction. The maximum time where $∆e_{t}^{*}\leq ∆e_{t}$ is the stability time at a particular thickness deviation $∆e_{t}^{*}$. Table 2 shows the material properties utilized in the simulations. It is worth noting that case 2 was considered as the reference process parameters.

.

## 3. Results and Discussion

In this section, the CFD model is used to assess the impact of process parameters, viz., fluid viscosity, traveling speed, traveling speed ratio, travel distance, and printed layer thickness, on the settling times required to achieve surface deviations of 2 μm, 2.5 μm, and 3 μm.

### 3.1. Fluid Viscosity

**Figure 4:**
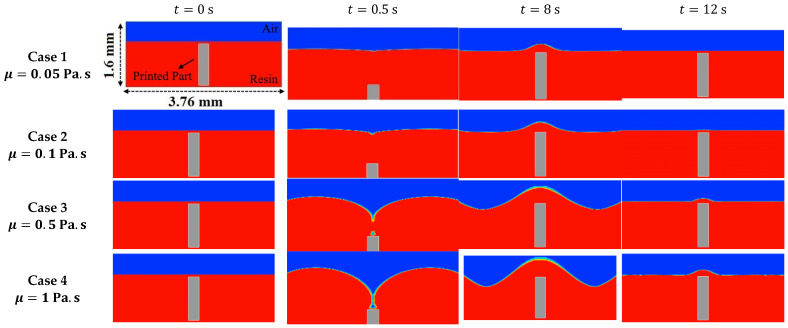
Contours of the fraction of fluid at four time steps and different fluid viscosity (TS=1.5 mm/s,TSR=1,TD=6 mm).

Figure 4 represents the fluid fraction contours at various instances for four different fluid viscosities given in cases 1 to 4. Initially, as the part moves down, the free surface becomes deformed, causing a downward curve at the center, as seen at *t* = 0.5 s. At lower viscosities of μ=0.05−0.1 Pa.s, the deformation of the free surface is marginal, whereas, at higher viscosities, the deformation of the free surface is profound. Subsequently, as the part moves upward, the shape of the free surface changes as the resin level rises above its initial level in the vicinity of the part, as shown at *t* = 8 s. The rise in liquid level is observed to be significant at all the values of viscosity. At higher viscosities, the rise in liquid level at the center is so high that the free surface has a wavy shape. As the part becomes stationary, the liquid resin gradually settles down, as seen at *t* = 12 s. The part must be held at this position until the free surface of the liquid is within the required limits for favorable printing, which is referred to as the settling time. Curing of the next layer before the settling time will result in nonuniform layer thickness (see Figure 4 at *t* = 12 s), leading to poor printing characteristics. Thus, optimizing the settling time is crucial in increasing the printing speed.

**Figure 5:**
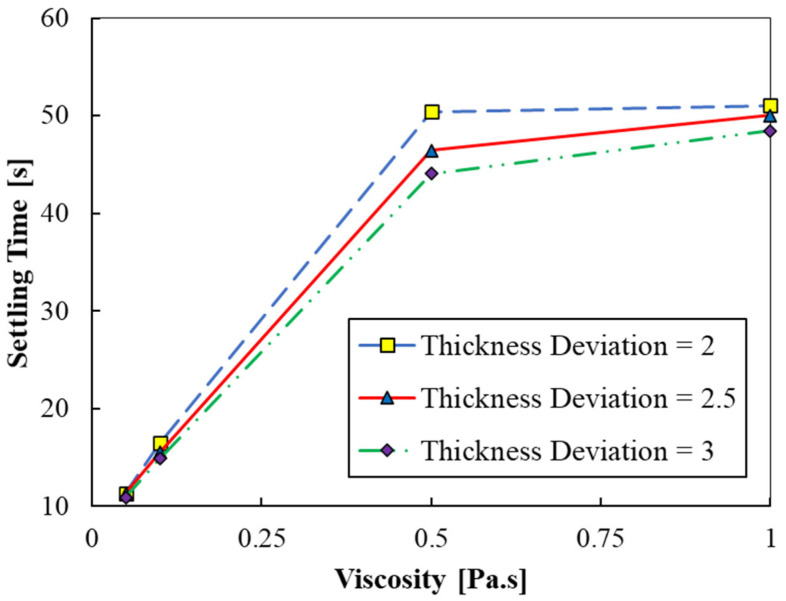
Settling time versus viscosity (cases 1 to 4).

Simulations were carried out for cases 1–4 of Table 2 to assess the effect of resin’s viscosity on the settling time. Figure 5 shows the variation of settling times $\left(t_{s}\right)$ with the resin’s viscosity (μ), plotted for three different free surface deviations $\left(∆e_{t}\right)$. It is observed that the settling times are very short at lower viscosities, and increase by an order of magnitude for a viscosity of 0.5 Pa.s. For highly viscous resins (above 0.5 Pa.s), the variation in settling times is marginal. The results indicate that the settling time is highly based on the resin’s viscosity, and lower viscosities are favorable for achieving higher printing speed and precision. In general, viscosity of the resin can be lowered by diluting it with several additives. This can help in achieving the desired printing speeds and print quality.

### 3.2. Traveling Speed

**Figure 6:**
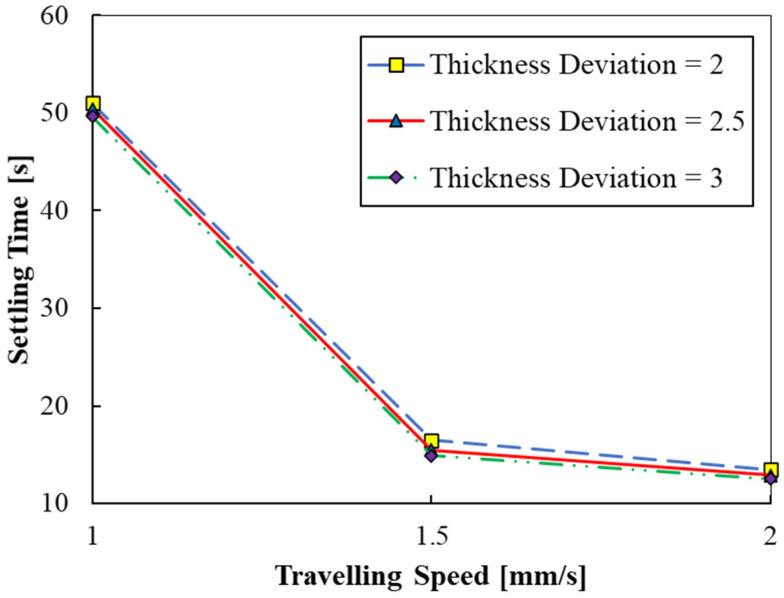
Settling time versus travelling speed (cases 2, 5, and 6).

Simulations corresponding to cases 2, 5, and 6 were carried out to analyze the effect of travelling speed on settling time for three different free surface deviations (see Figure 6). The results reveal that higher traveling speeds lead to shorter settling times. The traveling speed of the build part was varied between 1 mm/s to 2 mm/s. The settling time decreases with an increase in the travelling speed. Between 1–1.5 mm/s, the settling time drops sharply, while there is only a marginal variation between 1.5–2 mm/s. While higher travelling speeds may induce turbulent effects, lower travelling speeds require a longer delay in the printing due to longer settling times. Therefore, an intermediate value of 1.5 m/s of traveling speed might be optimal for better print quality and faster printing. In addition, the variation in the three settling times for the three different conditions are within 4 s, suggesting that higher part accuracies can be achieved with a slight increase in the delay time between the part movement and the UV curing.

It should be noted that the difference between traveling time and final time represents the actual stability time. The actual stability time reduces (39, 8.5, and 7.5 s) by incrementing TS from 1 to 2 mm/s, respectively.

### 3.3. Travelling Speed Ratio

**Figure 7:**
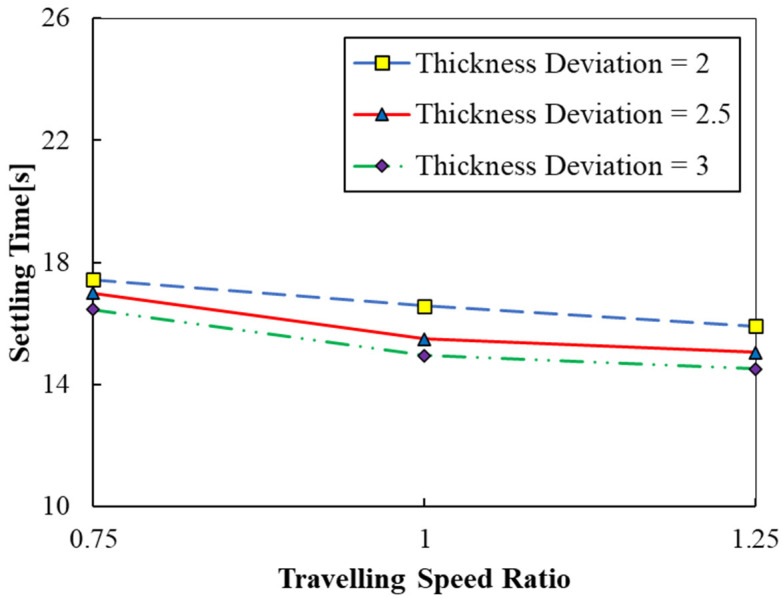
Settling time versus travelling speed ratios (cases 2, 7, and 8).

Normally, a trade-off is represented by additive manufacturing technologies between quality and printing speed. Based on Equation (4), the ratio of the up-to-down traveling speeds of the build part is represented by the dimensionless parameter:

(4) $$TSR=\frac{S_{up}}{S_{down}}$$

where S_up_ and S_down_ are the upward and downward traveling speeds, respectively. The effects of travelling speed ratio (0.75 ≤ TSR ≤ 1.25) on the settling time using the parameters of case 2 are presented in Figure 7. According to Figure 7, by increasing the TSR from 0.75 to 1.25, the settling times are observed to decrease. The decrease in settling times is observed to be higher when the TSR is increased from 0.75 to 1, as compared to 1 to 1.25. The results reveal that the upward speed of the part must always be more than or equal to its downward speed. Since there is only a marginal variation between the settling times at TSR = 1 and TSR = 1.25, using the same upward and downward speeds is a reasonably good choice. Furthermore, it is observed that the variation in settling times with TSR is very small compared to the variation with resin viscosity and travelling speed. Therefore, it is inferred that TSR is an insignificant parameter in the context of the settling time.

### 3.4. Print Layer Thickness

**Figure 8:**
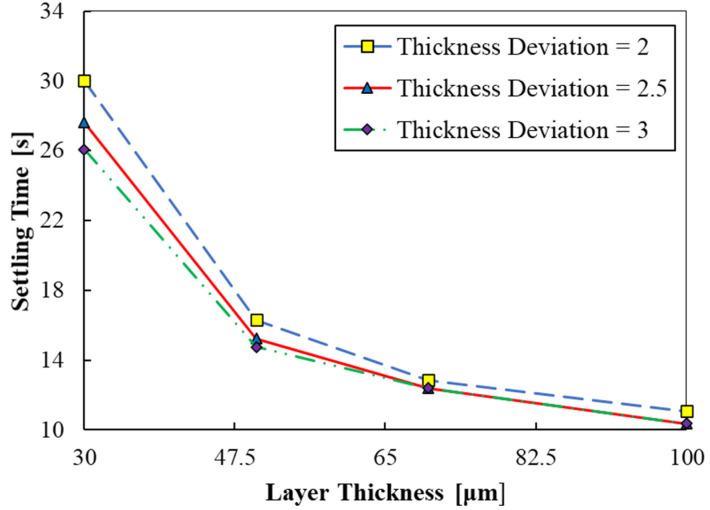
Settling time versus print layer thickness (cases 2, 9 to 11).

The settling times obtained at different print layer thicknesses (30, 50, 70, and 100 μm) corresponding to cases 2 and 9 to 11 are plotted in Figure 8. The print layer thickness also dictates the strength and accuracy of the part. Smaller print layers have higher strength and better part accuracy, even though the printing speeds are lower. Figure 8 shows that settling times are very high for smaller values of print thickness. The variation in the data of settling time with print layer thickness is akin to an exponential decay. The settling time nearly reduces by half with an increase in print layer thickness from 30 to 47.5 μm. Thereafter, the settling time gradually decreases with an increase in print layer thickness, wherein about a 25% drop in settling time is observed as the print layer thickness is nearly doubled from 47.5 to 100 μm.

The quality and speed of each print are affected by layer thickness. The printing speed is determined by the number of layers required for creating an object, thus, the printing time needed. By the lower layer thickness, it takes longer to create a 3D-printed object with a given height. A lower layer thickness indicates that the printer should print further layers to obtain the same total height. Thus, a much slower print is achieved. However, by the lower thickness, a better print quality is obtained. It is interesting to observe that printing thinner layers will require higher delay times due to higher settling times, thereby leading to a further increase in the printing times. Therefore, to achieve higher part precision and quality, printing time would exponentially increase. A trade-off between part quality and productivity can be achieved by an appropriate choice of the print layer thickness. As depicted in Figure 8, a descending pattern was revealed by the stability time by augmentation of the printed layer thickness from 30 to 100 μm. In addition, at a layer thickness of 100 μm, the minimum stability time was found ($t_{s}=$10.5 s).

### 3.5. Travel Distance

**Figure 9:**
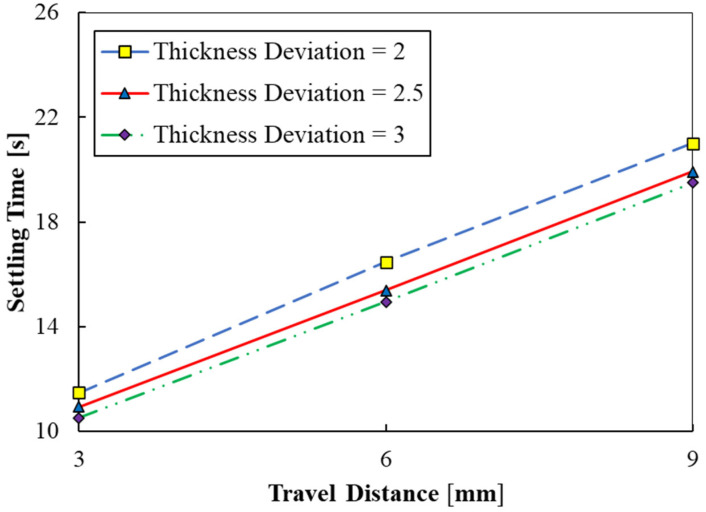
Settling time versus travel distance considering cases 2, 12, and 13.

Travel distance refers to the distance covered by the part as it moves down and up before each layer is printed. The variation in settling time with travel distance of the part is shown in Figure 9, for parameters shown in cases 2, 12, and 13. As can be seen in Figure 9, the settling time linearly increases with an increase in the travel distance from 3 to 9 mm. The settling times are about 10–12 s at a travel distance of 3 mm, whereas it increases to 19–21 s at a travel distance of 9 mm. The results indicate that longer travel distances could be avoided for increasing the printing speed as they require longer settling times. This could be because longer travel distances could lead to disturbance in the resin over a greater volume. Consequently, it would require greater settling time for the resin to come to a standstill. It is inferred that travel distance is also a significant parameter that influences the settling time.

## 4. Conclusions

The present work deals with CFD simulations of top-down DLP-based 3D printing. The present paper aimed to assess the influences of different printing parameters, including fluid viscosity, travelling speed, travelling speed ratio, travel distance, and printed layer thickness. The following findings were obtained from the present research:

1. By increasing the fluid viscosity from 0.05 to 1 Pa.s, the fluid interface will need more time for reaching a stable state. According to the plotted working curves, stabilizing the fluid interface requires approximately 16.5 s when applying the reference parameters.
2. Considering the optimization results, for case 4 with the fluid viscosity of 1 Pa.s, a maximum stability time of 51 s was achieved.
3. A diminishing trend was found for the stability time by augmentation of the traveling speed from 1 to 2 mm/s, remarkably. Moreover, the maximum stability time of almost 51 s was obtained for thickness deviation of 2 µm and the traveling speed of 1 mm/s.
4. A smaller stability time of the fluid interface was obtained by increasing the travelling speed ratio from 0.75 to 1.25. In addition, the minimum and maximum stability times for the travelling speed ratio parameter considering thickness deviation of 2.5 µm were obtained at roughly 15 s and 17 s, respectively.
5. A stable situation was obtained for the fluid interface in a shorter time considering high printed layer thickness values.
6. According to the results of the travel distance parameter, the minimum and maximum stability times at thickness deviation of 3 µm were achieved at approximately 10.5 s and 19.5 s, respectively.

## Figures and Tables

**Table 1:** Mesh quality and resolution.

Quantity	Unit	Mesh 1	Mesh 2	Mesh 3
Number of cells	-	33,108	68,580	104,091
Maximum cell size (in X/Z direction)	μm	31.8/49	20.8/37.6	17.30/30.50
Minimum cell size (in X/Z direction)	μm	30.1/13.6	20.8/7.5	16.66/5.99
Maximum ratio of adjacent cell size ratio (in X/Z direction)	-	1.02/1.22	1/1.23	1/1.20
Maximum aspect ratio (X:Z ratio)	-	2.33	2.77	2.88

**Table 2:** Reference material and processing properties.

Case Numbers	$\mathrm{Density}\;(kg/m^{3})$	Viscosity(Pa.s)	Travelling Speed(mm/s)	Travelling Speed Ratio (-)	Layer Thickness(μm)	Travel Distance(mm)
Case 1	1100	0.05	1.5	1	50	6
Case 2		0.1	1.5	1	50	6
Case 3		0.5	1.5	1	50	6
Case 4		1	1.5	1	50	6
Case 5		0.1	1	1	50	6
Case 6		0.1	2	1	50	6
Case 7		0.1	1.5	0.75	50	6
Case 8		0.1	1.5	1.25	50	6
Case 9		0.1	1.5	1	30	6
Case 10		0.1	1.5	1	70	6
Case 11		0.1	1.5	1	100	6
Case 12		0.1	1.5	1	50	3
Case 13		0.1	1.5	1	50	9

## Data Availability

Data is available on request.

## Nomenclature

Symbols	
$e_{t}$	The thickness of the layer at time t
$e_{n}$	Nominal thickness of the layer
$u_{i}$	The velocity component in the i-direction
p	Local pressure
$g_{i}$	Gravitational body forces per mass unit in the i-direction
t	Time
$t_{m}$	Traveling time
$t_{f}$	Finishing time
$t_{s}$	Stability time
$x_{i}$	Spatial coordinates
x,y,z	Coordinates
Greek symbols	
$\delta_{i}$	Distance of the fluid interface from the surface of the build part
$∆e_{t}$	Thickness deviation
$∆e_{t}^{*}$	Particular thickness deviation
μ	Fluid viscosity
Abbreviations	
AM	Additive manufacturing
CFD	Computational fluid dynamics
DLP	Digital light processing
DMD	Digital micromirror device
2D	Two-dimensional
3D	Three-dimensional
FDM	Fused deposition modeling
GMRES	Generalized minimum residual
LCD	Liquid crystal display
SLA	Stereolithography
TD	Travelling distance
TS	Traveling speed
TSR	Traveling speed ratio
UV	Ultraviolet
VOF	Volume of fluid
